# Comparison of Ilizarov Bifocal, Acute Shortening and Relengthening with Bone Transport in the Treatment of Infected, Segmental Defects of the Tibia

**DOI:** 10.3390/jcm9020279

**Published:** 2020-01-28

**Authors:** Irene K. Sigmund, Jamie Ferguson, Geertje A.M. Govaert, David Stubbs, Martin A. McNally

**Affiliations:** 1 The Bone Infection Unit, Nuffield Orthopaedic Centre, Oxford University Hospitals, Foundation NHS Trust, Windmill Rd, Headington, Oxford OX3 7HE, UK; irene.sigmund@meduniwien.ac.at (I.K.S.); Jamie.Ferguson@ouh.nhs.uk (J.F.); David.Stubbs@ouh.nhs.uk (D.S.); 2 Department of Orthopaedics and Trauma Surgery, Medical University of Vienna, Spitalgasse 23, Vienna 1090, Austria; 3 Department of Trauma Surgery, University of Utrecht, University Medical Centre Utrecht (UMCU), 3512 Utrecht, The Netherlands; g.a.m.govaert@umcutrecht.nl

**Keywords:** infection, tibia, Ilizarov, bifocal, acute shortening, distraction, bone transport, non-union, outcome

## Abstract

This prospective study compared bifocal acute shortening and relengthening (ASR) with bone transport (BT) in a consecutive series of complex tibial infected non-unions and osteomyelitis, for the reconstruction of segmental defects created at the surgical resection of the infection. Patients with an infected tibial segmental defect (>2 cm) were eligible for inclusion. Patients were allocated to ASR or BT, using a standardized protocol, depending on defect size, the condition of soft tissues and the state of the fibula (intact or divided). We recorded the Weber–Cech classification, previous operations, external fixation time, external fixation index (EFI), follow-up duration, time to union, ASAMI bone and functional scores and complications. A total of 47 patients (ASR: 20 patients, BT: 27 patients) with a median follow-up of 37.9 months (range 16–128) were included. In the ASR group, the mean bone defect size measured 4.0 cm, and the mean frame time was 8.8 months. In the BT group, the mean bone defect size measured 5.9cm, and the mean frame time was 10.3 months. There was no statistically significant difference in the EFI between ASR and BT (2.0 and 1.8 months/cm, respectively) (*p* = 0.223). A total of 3/20 patients of the ASR and 15/27 of the BT group needed further unplanned surgery during Ilizarov treatment (*p* = 0.006). Docking site surgery was significantly more frequent in BT; 66.7%, versus ASL; 5.0% (*p* < 0.0001). The infection eradication rate was 100% in both groups at final follow-up. Final ASAMI functional rating scores and bone scores were similar in both groups. Segmental resection with the Ilizarov method is effective and safe for reconstruction of infected tibial defects, allowing the eradication of infection and high union rates. However, BT demonstrated a higher rate of unplanned surgeries, especially docking site revisions. Acute shortening and relengthening does not reduce the fixator index. Both techniques deliver good functional outcome after completion of treatment.

## 1. Introduction

Infected segmental defects are common after tibial injury [1,2,3]. They are problematic for patients, due to the prolonged treatment time, permanent functional deficits and high reinfection and non-union rates [4]. Although there has been an improvement in the treatment of open fractures since the introduction of management guidelines, advanced implant designs and less traumatic surgical techniques, the infection rate is still high, ranging from 9% to 18% [5,6,7,8,9,10]. Furthermore, recurrence of infection is common, affecting 10% to 20% of patients [11,12,13,14]. A structured approach, with adequate debridement, excision of necrotic tissue, antimicrobial therapy and reconstruction of the bone and soft tissues, is of immense importance to eradicate infection and restore function [15,16,17]. To manage the resulting bone defects, distraction osteogenesis techniques such as Ilizarov acute shortening with bifocal relengthening (ASR) and bone transport (BT) have been shown to be effective treatment options to achieve union and infection clearance [1,12,15,16,18]. This technique permits not only the gradual restoration of the bone, but also deformity correction, correction of joint contractures, and rehabilitation, with early weight bearing to avoid muscle wasting and disuse osteopenia.

Generally, each surgeon decides on the method of distraction histogenesis (ASR vs. BT), based on clinical features and personal experience [19,20,21,22]. Small bone defects are commonly treated by acute shortening, with or without relengthening, while in large bone defects a BT is often preferred. However, no standardized or validated protocol has been described in the literature and it is often impossible to determine why a particular method has been chosen.

In this study, we compared acute shortening and relengthening with bone transport for the reconstruction of segmental defects in a consecutive, prospectively analysed series of complex infected tibias, by using a standardized protocol. We evaluated the treatment duration, outcome and complications during and after the procedures.

## 2. Materials and Methods

### 2.1. Study Design

This prospective study was conducted in a tertiary healthcare centre, providing advanced specialty care (orthopaedic surgeons, plastic surgeons, specialist Ilizarov nurses and physiotherapists and infectious disease physicians specialized in musculoskeletal infections) to patients with musculoskeletal infections. The study was performed in accordance with the Declaration of Helsinki and approved by the hospital governance board (ID 5778).

### 2.2. Study Population

Consecutive patients over 18 years old, who had a segmental bone defect after surgery for infected non-union or osteomyelitis of the tibia were included. Information on demographics, comorbidities, associated deformities, imaging, histological and microbiological results from intraoperative sampling, surgical treatment procedures, and complications were recorded. 

Preoperatively, each patient was reviewed by a multidisciplinary team (orthopaedic and plastic surgeons, infectious disease physicians, and a specialist Ilizarov nurse practitioner) who agreed the treatment plan. The classification of Weber and Cech [23], was determined at operation, based on the non-union viability as defined according to the vascularity of the bone. 

The following outcome parameters were evaluated after surgery: recurrent infection, total frame time, external fixator index in months/cm (EFI), complications, the Association for the Advancement of Methods of Ilizarov (ASAMI) bone and functional classification scores, and further unplanned surgery (defined as any additional surgery undertaken after frame removal). 

### 2.3. Infection Definition

An infected segmental tibial bone defect was diagnosed according to the International Consensus criteria for fracture-related infections (FRI) [24,25]. Infection was present when at least one of the following criteria applied: (i) a draining sinus, (ii) an abscess or intraoperative purulence, (iii) significant growth of a microorganism from intraoperative tissue sampling (at least two or more positive sterile site cultures with indistinguishable organisms), and (iv) positive histopathology supportive of deep active infection.

### 2.4. Tissue Sampling and Surgical Procedures

All patients were treated with a single operation comprising an excision of the infected dead bone segment, application of the Ilizarov fixator, corticotomy and skin closure (including free tissue transfer, if needed). Antimicrobial treatment was stopped for at least 14 days before surgery. A tourniquet was used in all patients. In the first step, the collection of specimens was performed as described previously [26].

For histopathological analysis, at least two deep tissue specimens were collected. If a sinus was present for more than five years, it was excised and sent for histological review to exclude squamous carcinoma. 

In the second step, the infected area was fully exposed to allow adequate debridement. Protecting the neurovascular structures, a segmental resection was performed to eradicate the non-viable zone. A careful resection of indurated subcutaneous tissue, infected and necrotic soft tissue, and necrotic bone was performed in all cases. Thereafter, the wound was manually washed with generous volumes of 0.05% aqueous chlorhexidine at low pressure. Without releasing the tourniquet, the pattern of bone bleeding was observed. If punctate bleeding was absent, a further resection was undertaken. 

### 2.5. Standardized Treatment Protocol

The choice of Ilizarov method was made depending on the criteria outlined below. An Ilizarov ring fixator was used in all cases (Smith and Nephew, Memphis, TN, USA). 

**Figure 1:**
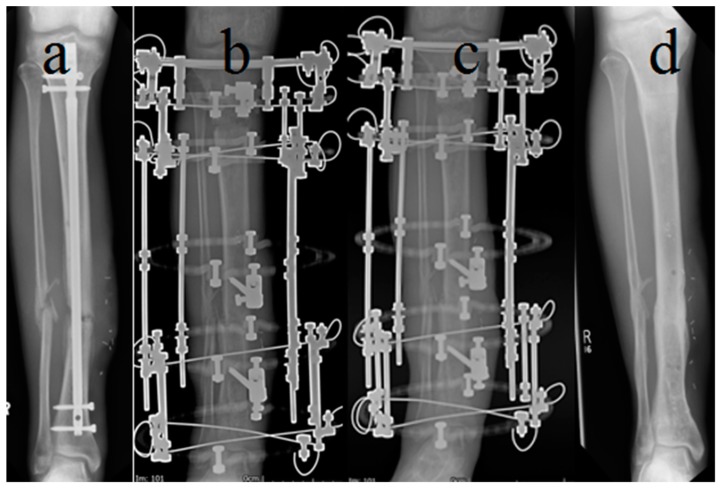
*Acute shortening with relengthening.* (**a**) Open fracture of tibia treated with early debridement, IM nailing and flap cover. Presented at three months, with a discharging sinus and exposed medial tibial bone. (**b**) A 2.5 cm resection was required. The tibia was acutely shortened to good bone contact and the flap reused to cover the defect. (**c**) Distraction was performed at 1 mm per day through the proximal corticotomy (**b**) and continued until equal leg length was achieved (at four weeks after surgery). (**d**) Eight months after surgery, the tibia was solidly healed with good alignment and no evidence of infection.

Acute shortening and relengthening (Figure 1) was performed in patients with bone defects of up to 5 cm, where it could be performed safely without soft tissue or neurovascular compromise. Segmental bone resection was performed to allow good bone contact with good alignment at the docking site. A corticotomy was performed at a separate metaphyseal location outside the infected zone. After 6–7 days latent period, length was gradually restored by distraction through the corticotomy site at 1 mm per day. Lengthening was continued until equal leg length was restored. The docking site was compressed at 0.5 mm per week for 2–3 weeks. 

**Figure 2:**
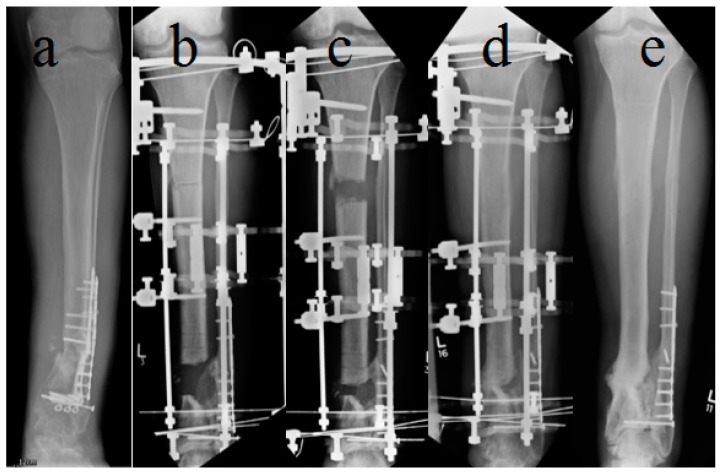
*Bone Transport.* (**a**) Previous open fracture of distal tibia, treated with internal fixation. The wound broke down and he was referred with a mobile infected non-union. Preoperative AP x-ray shows that the lateral tibial plate has broken at the non-union. (**b**) Four centimetre resection of the non-union after removal of the infected tibial plate. The fibula was intact and well aligned, so a bone transport was preferred. (**c**) Twenty-six days after surgery. The proximal corticotomy is distracted at 1 mm per day. (**d**) Six weeks after docking. No surgery was required to secure union at the docking site. (**e**) Four months after frame removal with well consolidated docking site, regenerate and good alignment.

Bone transport (Figure 2) was selected in all patients with bone defects larger than 5 cm or when the fibula was intact and well aligned. In some patients, with defects <5 cm, the soft tissues were heavily scarred and indurated due to chronic infection. If attempted, acute shortening caused vascular compromise in the foot, thus bone transport was elected as the reconstruction method. Corticotomy, latent period and distraction rate were the same as in the ASR group. When docking was achieved in the BT group, no elective surgery on the docking site was planned.

Skin closure was performed in all cases. If a direct wound closure was possible, early mobilization was commenced, and full-weight-bearing encouraged on day two. If a local or free microvascular muscle flap was used to restore a healthy soft tissue envelope, full weight bearing was allowed after flap healing based on the plastic surgeon’s discretion (usually after seven days). 

Empiric antibiotics (Vancomycin and Meropenem) were administered intraoperatively, after sampling. According to the susceptibility of the isolated pathogen(s), the antimicrobial treatment was adjusted by the infectious disease specialist team and continued for at least six weeks. 

### 2.6. Statistical Analysis

Continuous variables are expressed as mean and range; categorical variables are described as absolute and relative frequencies (percentage). Student’s *t*-test and Fisher’s exact test were performed to compare metric and binary variables between the distributions of both groups. A chi-squared test was used to compare the overall complication rate between both groups. All estimated parameters are reported with 95% confidence intervals. The significance level for all tests was 5% (*p* < 0.05). The software package XLSTATPM (version 2017; XLSTAT; Addinsoft, New York, NY, USA) was used for statistical analysis.

## 3. Results

### 3.1. Demographics and Infection Characteristics

Forty-seven patients with an infected tibial segmental defect were included. Of these, 20 (43%) patients were treated with ASR and 27 (57%) with BT. In eight cases with bone defects under 5 cm, ASR was not possible because of scarred soft tissue or neurovascular compromise after attempted shortening. Demographic data and surgical features of the 47 analysed cases are shown in Table 1. Median follow-up was 37.9 months (range 16–128). 

Six (13%) patients presented with muscle flaps in place, which were “reused” (ASR [*n* = 3], BT [*n* = 3]). In a further 11 (23%) patients, a new muscle flap was required to allow soft tissue closure. Of these, eight (73%) had a free gracilis muscle flap (ASR [*n* = 4], BT [*n* = 4]), two (18%) a free latissimus dorsi muscle flap (BT group), and one (9%) a local gastrocnemius muscle flap (BT group). There was no difference in the need for new free flap reconstruction between the groups (ASR, 4/17; BT, 7/24 *p* = 1.000).

The most common causative microorganism was *Staphylococcus aureus*, followed by coagulase negative staphylococci and *Pseudomonas aeruginosa*. 

### 3.2. Outcome

No patient suffered a recurrent FRI during follow-up (Table 2). 

The initial union rate was 18/20 (90%) for ASR and 17/27 (63%) for BT. This difference was statistically significant (*p* = 0.047). All non-union cases were infection free and healed after further fixation (see below). Therefore, the final union rate was 100% in both groups. 

At the end of treatment, no patient had a significant leg length discrepancy (>1 cm) or angular or rotational deformity (>5 degrees). 

**Figure 3:**
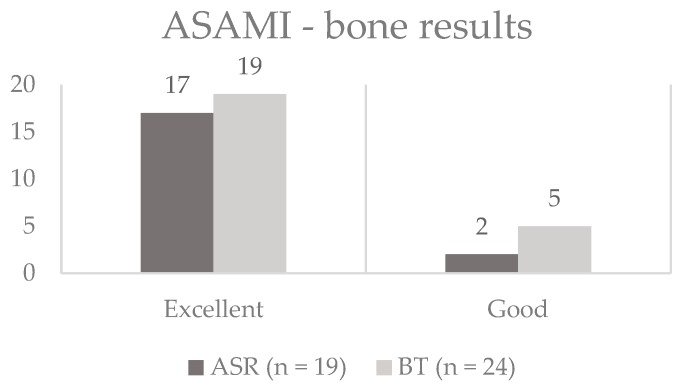
Results of the Association for the Advancement of Methods of Ilizarov (ASAMI) bone classification in the acute shortening/relengthening (ASR) and bone transport (BT) group. There was no statistically significant difference between the groups (*p* = 0.682). The y-axis gives the absolute numbers.

Figure 3 illustrates the final ASAMI bone classification results for each group. The ASAMI bone scores showed no significant difference between both groups at the end of treatment (*p* = 0.682).

**Figure 4:**
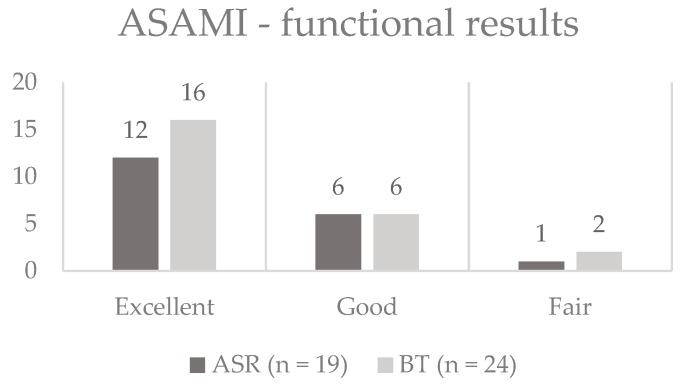
Results of the ASAMI functional classification in the acute shortening/relengthening (ASR) and bone transport (BT) group. There was no statistically significant difference between the groups (*p* = 0.705). The y-axis gives the absolute numbers.

Figure 4 shows the final ASAMI functional classification results. There was no significant difference between both groups regarding ASAMI functional score (*p* = 0.705).

### 3.3. Unplanned Surgeries during Fixator Time

Eighteen (38%) unplanned surgeries (including docking site procedures) had to be performed in both groups. Of these, 15 (*n* = 15/27; 56%) were needed in the BT group with only three (*n* = 3/20; 15%) surgeries in the ASR group. There was a statistically significant difference between groups (*p* = 0.006). Thirteen of the 15 (87%) patients with an unplanned procedure in the BT group had an operation at the docking site. All surgeries are summarized in Table 3. 

If the docking site procedures were excluded, no statistically significant difference was found between the ASR and BT group (*p* = 0.638). 

### 3.4. Unplanned Surgeries after Fixator Removal

After the removal of the Ilizarov frame, thirteen (*n* = 13/47; 28%) unplanned reoperations were needed in both groups. In the ASR group only three (*n* = 3/20; 15%) further revision surgeries were required with ten (*n* = 10/27; 37%) in the BT group. However, no significant difference was found (*p* = 0.114). Overall, docking site surgery was significantly more frequent in BT; 66.7%, versus ASL; 5.0% (*p* = 0.0001). A detailed list of revision surgeries after the removal of the Ilizarov frame is shown in Table 3.

In total, 31 unplanned surgeries were needed during the whole observation period, six (30%) in the ASR group and 25 (93%) in the BT group. This difference was statistically significant (*p* = 0.013).

### 3.5. Other Complications

During treatment, 22 patients (47%) suffered at least one superficial pin infection requiring treatment with oral antibiotics. These were equally common after ASR or BT. One patient had the removal of a pin due to persistent pin site infection, which was resolved after removal. One patient had a release of painful tethered skin around a pin and two bone transport patients needed repeat corticotomy due to premature fusion of the lengthening site. There were no other significant complications observed.

## 4. Discussion

This series confirms that segmental excision is an effective method of eradicating infection, with no patient suffering a recurrent infection at follow-up. Resection of an entire segment of bone allows the complete removal of all dead bone and biofilm, and so will give a better chance of eradication compared to an intralesional excision. Outcomes may also be improved by the multidisciplinary approach from the specialist bone infection team, with careful attention to diagnostics, surgical management, soft tissue reconstruction and antimicrobial therapy. 

Few studies have compared the outcome of acute shortening and relengthening with bone transport by using Ilizarov techniques for an infected tibial non-union with a segmental bone defect [19,20,22] (Table 4). To the best of our knowledge, this series represents the largest single-centre prospective study with a standardized treatment protocol. In previous studies, the surgical treatment was chosen based on clinical parameters according to the surgeon’s preference, and the reason for each decision is usually not described. This makes it very difficult to give any recommendation on which cases would be best treated by any particular technique.

In this study, we had a clear protocol for the choice of Ilizarov method, allowing a better comparison of the techniques in cases where they may be more appropriately applied. Also, we limited the variability between groups by using the same fixator type (Ilizarov rings), same sampling method and diagnostic criteria, same antibiotic regime and same latent period before distraction. It has been shown that different fixator constructs may affect the outcome of the Ilizarov method [27].

Acute shortening and relengthening using an Ilizarov ring fixator had a higher initial union rate (90% vs. 63%) and a much lower rate of unplanned surgery during treatment (30% vs. 93%) compared to bone transport. The majority of unplanned operations were docking site procedures. Our rate of 67% docking site surgery in the bone transport group is similar to that reported by Tetsworth, et al. [22] (67%) and Eralp, et al. [19] (62%), but our interventions in the ASR group were much less frequent, at 5%. Tetsworth, et al. [22] operated on 38% of ASR cases and suggested that there may be a place for primary bone grafting of acutely shortened docking sites. We do not agree with this. In his series, Tetsworth, et al. included patients with defects larger than 5 cm in the ASR group. These patients could not be acutely shortened and so had a partial shortening followed by a gradual shortening to bone contact. This is analogous to a short bone transport and may account for the higher rate of docking site surgery. We agree that ASR is a technically more demanding procedure than bone transport. It is often difficult to achieve acute shortening due to swelling in the soft tissues around the defect. Displacement of the tissues may prevent accurate apposition of the bone ends with good alignment. Because of these concerns, we recommend that ASR should be reserved for defects under 5 cm, to allow patients to receive the benefits of the technique. 

It has been suggested that the routine freshening and bone grafting of docking sites should be performed [28,29,30]. However, in our series and others, this would result in the overtreatment of one third of cases. We therefore do not recommend routine docking site surgery, but the decision to operate should not be delayed for a prolonged period. If there is no clear sign of bridging callous across the docking site within eight weeks of docking, we advocate surgery. In this series, we usually freshened the docking site and provided bone grafting if there was a defect present. We only advocate internal fixation if the stability of the fixator has been lost. 

Unfortunately, ASR did not reduce the total time in treatment compared to BT. The duration of treatment is determined by three phases. The latent period (from corticotomy to the onset of distraction) was the same in both groups. The lengthening phase and the consolidation phase (from completion of relengthening or docking to frame removal) are mainly determined by the length of the segment to be reconstructed, and therefore are unlikely to be affected by the choice of technique. In most cases, docking site healing occurred (with or without adjunctive surgery) before consolidation of the regenerate, and so did not delay treatment time in the bone transport group.

We were also concerned that acute shortening may defunction muscles crossing the ankle joint, making early mobilisation more difficult, with a potentially reduced functional outcome. This was not observed. Patients in both groups mobilised full-weight-bearing, beginning as soon as the soft tissues allowed. In our study, the majority of patients showed excellent or good bony and functional results after treatment. This is in line with the reported data in the literature [19,20,22] (Table 4). Clearly, both techniques allow good functional recovery, but this is only achieved with careful attention to rehabilitation with a guided physiotherapy program, supervised by therapists who are familiar with Ilizarov methods. 

Numerous methods have been described for the restoration of bone in segmental tibial defects, including cancellous bone grafting, the implantation of bone substitutes, Papineau technique, non-vascularised and vascularised bone grafts and the induced membrane technique [31,32,33,34,35]. All have reported successful results, but treatment times are long and reoperation rates are high. In recent literature reviews comparing defect management methods, there was no clear advantage of one technique over another, either in outcome or treatment time [36,37]. Ilizarov methods do have an advantage as they can address concomitant deformity and leg length difference more easily.

At our institution, the soft tissue closure is always performed in the same operation as the Ilizarov procedure as a single stage operation. This requires long operative times but reduces the number of visits to theatre and the overall time in treatment. We have shown that it is successful in this series and in cases of chronic osteomyelitis [38]. In the tibia, with chronic infection, up to one third of patients may require tissue transfer (local or free flap) to achieve closure. This requires meticulous preoperative planning between the orthopaedic and plastic surgeon [17]. Often, frame application may need to be modified to allow access to microvascular anastomosis for free flaps. However, we have shown that it is safe to transfer free flaps and reuse existing flaps in combination with distraction osteogenesis [39]. In this study, no patient treated with a flap had a wound-healing disorder or failure of the flap.

A limitation of the study is the small sample size in both groups. Nevertheless, it represents the largest prospective single-centre study in adults comparing ASR and BT, and is sufficiently capable of comparing the techniques [22]. Another concern is the difference in the segment length of the two groups. This was dictated as part of the protocol and was due to the pragmatic difficulty of acute shortening of long defects with scarred and indurated soft tissues. It may be appropriate to consider a randomised trial of ASR versus BT for defects up to 5 cm where shortening is possible, to address this. However, the sample size would need to be large, as the differences in outcome and treatment parameters are likely to be small.

## 5. Conclusions

Bone transport using the Ilizarov technique is a less demanding procedure, but showed a higher rate of unplanned surgeries, especially docking site revisions. Almost all patients having a bone transport required an unplanned procedure. While acute shortening and relengthening is technically more difficult, (fibula osteotomy, soft tissue dissection, increased risk of neurovascular injuries, etc.), less revision surgeries are needed. ASR is limited by neurovascular compromise, while BT is also applicable in large bone defects, with no need to expose the fibula. Nevertheless, both methods are safe and effective distraction osteogenesis techniques for the treatment of infected tibial non-unions with similar functional and bony outcomes. 

## Figures and Tables

**Table 1:** Demographic data and surgical features of the study cohort stratified in groups with acute shortening and relengthening (ASR) and bone transport (BT). Values in bold are statistically significant.

Demographic Data	ASR (*n* = 20)	BT (*n* = 27)	*p*-Value
Patient age (mean)	48.9 (21–78)	50.9 (29–75)	0.738
Bone Defect (cm)	4.0 (2–5)	5.9 (3–10)	0.007
Prior Surgeries per patient	2.9 (1–5)	2.7 (1–6)	0.858
Sinus tract	8	9	0.761
Muscle flap	7	10	1.000
EF time (months)	8.8 (5–16)	10.3 (7–17)	0.064
EFI (months/cm)	2.0 (1.3–2.8)	1.8 (0.9–2.7)	0.223
Weber–Cech type (*n*[%])			
Type C	1 (5)	0 (0)	
Type D	4 (20)	4 (15)	
Type E	3 (15)	5 (19)	
Type F	12 (60)	18 (67)	

A statistically significant difference between both study groups was demonstrated for the magnitude of bone defect (*p* = 0.007) with larger defects in the BT group. The absolute external fixator time was shorter in the ASR group, but it was not statistically significant (*p* = 0.064).

**Table 2:** Outcome of the treatment of infected tibial segmental defect in the acute shortening/relengthening (ASR) group and the bone transport (BT) group.

	Total (*n* = 47)	ASR (*n* = 20)	BT (*n* = 27)	*p*-Value
Infection-free	47 (100)	20 (100)	27 (100)	1.000
Union without further surgery	35 (74)	18 (90)	17 (63)	0.047
Unplanned Reoperation during EF treatment including docking site	18 (38)	3 (15)	15 (56)	0.006
Unplanned Reoperation during EF treatment excluding docking site	5 (11)	3 (15)	2 (7)	0.638
Unplanned Reoperation after EF removal	13 (28)	3 (15)	10 (37)	0.114
Refracture after EF removal	3 (7)	2 (11)	1 (4)	0.575
Final Infection-free union	47 (100)	20 (100)	27 (100)	1.000

**Table 3:** Surgeries during and after external fixator (EF) treatment in the acute shortening/relengthening (ASR) group and the bone transport (BT) group.

	Total	ASR	BT	*p*-Value
**During EF treatment**				
Fibular Division	1 (2)	1 (5)	0 (0)	0.426
Insertion of further pins	1 (2)	1 (5)	0 (0)	0.426
Tethered pin site release	1 (2)	1 (5)	0 (0)	0.426
Bone grafting only	4 (9)	0 (0)	4 (15)	0.126
Freshening of docking site	3 (6)	0 (0)	3 (11)	0.251
Docking Site Realignment	4 (9)	0 (0)	4 (15)	0.126
Re-Corticotomy	2 (4)	0 (0)	2 (7)	0.500
BMP only	2 (4)	0 (0)	2 (7)	0.500
**After EF removal**				
Plate only	4 (9)	0 (0)	4 (15)	0.126
Plating and bone grafting	1 (2)	1 (5)	0 (0)	0.426
Plating and BMP	1 (2)	0 (0)	1 (4)	1.000
EF reapplication	1 (2)	1 (5)	0 (0)	0.426
Intramedullary Nail	4 (9)	1 (5)	3 (11)	0.626
Ankle Fusion	2 (4)	0 (0)	2 (7)	0.500
**Total**	31	6	25	**0.013**

NOTE. The values given are the number of cases, with percentage in parentheses. Values in bold are statistically significant. EF = external fixator, BMP = Bone Morphogenic Protein.

**Table 4:** Results of comparative trials in the literature.

	Khan et al. [20]		Eralp et al. [19]		Testworth et al. [22]		Present Study	
Study design	prospective		retrospective		retrospective		prospective	
Number of patients	24		74		42		47	
Procedure	ASR (*n* = 16)	BT (*n* = 8)	ASR (*n* = 45)	BT (*n* = 29)	ASR (*n* = 21)	BT (*n* = 21)	ASR (*n* = 20)	BT (*n* = 27)
Mean bone defect (cm)	3.3 (in all patients)		5.9	5.3	5.8	7.0	4.0	5.9
Mean EF time (months)	8		9	10	10	13	8.8	10.3
Mean EFI (m/cm)	4.2		1.6	2.1	1.7	1.8	2.0	1.8
ASAMI functional	7 excellent 8 good 1 fair	1 excellent 4 good 1 fair 1 failure 1 died (advanced liver disease)	34 excellent 8 good 2 fair1 poor	22 excellent 3 good 4 poor	14 excellent 6 good 1 fair	14 excellent 6 good 1 fair	12 excellent 7 good 1 fair	19 excellent 6 good 2 fair
ASAMI bone	6 excellent 9 good 1 fair	5 good 2 poor 1 died	35 excellent 8 good 2 fair	21 excellent 4 good 4 poor	19 excellent 2 good	15 excellent 5 good 1 fair	18 excellent 2 good	22 excellent 5 good
